# MicroRNA-19b-3p Modulates Japanese Encephalitis Virus-Mediated Inflammation via Targeting RNF11

**DOI:** 10.1128/JVI.02586-15

**Published:** 2016-04-14

**Authors:** Usama Ashraf, Bibo Zhu, Jing Ye, Shengfeng Wan, Yanru Nie, Zheng Chen, Min Cui, Chong Wang, Xiaodong Duan, Hao Zhang, Huanchun Chen, Shengbo Cao

**Affiliations:** a State Key Laboratory of Agricultural Microbiology, Huazhong Agricultural University, Wuhan, Hubei, People's Republic of China; b Laboratory of Animal Virology, College of Veterinary Medicine, Huazhong Agricultural University, Wuhan, Hubei, People's Republic of China; c The Cooperative Innovation Center for Sustainable Pig Production, Huazhong Agricultural University, Wuhan, Hubei, People's Republic of China; d Key Laboratory of Development of Veterinary Diagnostic Products, Ministry of Agriculture, Huazhong Agricultural University, Wuhan, Hubei, People's Republic of China

## Abstract

Japanese encephalitis virus (JEV) can invade the central nervous system and consequently induce neuroinflammation, which is characterized by profound neuronal cell damage accompanied by astrogliosis and microgliosis. Albeit microRNAs (miRNAs) have emerged as major regulatory noncoding RNAs with profound effects on inflammatory response, it is unknown how astrocytic miRNAs regulate JEV-induced inflammation. Here, we found the involvement of miR-19b-3p in regulating the JEV-induced inflammatory response *in vitro* and *in vivo*. The data demonstrated that miR-19b-3p is upregulated in cultured cells and mouse brain tissues during JEV infection. Overexpression of miR-19b-3p led to increased production of inflammatory cytokines, including tumor necrosis factor alpha, interleukin-6, interleukin-1β, and chemokine (C-C motif) ligand 5, after JEV infection, whereas knockdown of miR-19b-3p had completely opposite effects. Mechanistically, miR-19b-3p modulated the JEV-induced inflammatory response via targeting ring finger protein 11, a negative regulator of nuclear factor kappa B signaling. We also found that inhibition of ring finger protein 11 by miR-19b-3p resulted in accumulation of nuclear factor kappa B in the nucleus, which in turn led to higher production of inflammatory cytokines. *In vivo* silencing of miR-19b-3p by a specific antagomir reinvigorates the expression level of RNF11, which in turn reduces the production of inflammatory cytokines, abrogates gliosis and neuronal cell death, and eventually improves the survival rate in the mouse model. Collectively, our results demonstrate that miR-19b-3p positively regulates the JEV-induced inflammatory response. Thus, miR-19b-3p targeting may constitute a thought-provoking approach to rein in JEV-induced inflammation.

**IMPORTANCE** Japanese encephalitis virus (JEV) is one of the major causes of acute encephalitis in humans worldwide. The pathological features of JEV-induced encephalitis are inflammatory reactions and neurological diseases resulting from glia activation. MicroRNAs (miRNAs) are small noncoding RNAs that regulate gene expression posttranscriptionally. Accumulating data indicate that miRNAs regulate a variety of cellular processes, including the host inflammatory response under pathological conditions. Recently, a few studies demonstrated the role of miRNAs in a JEV-induced inflammatory response in microglia; however, their role in an astrocyte-derived inflammatory response is largely unknown. The present study reveals that miR-19b-3p targets ring finger protein 11 in glia and promotes inflammatory cytokine production by enhancing nuclear factor kappa B activity in these cells. Moreover, administration of an miR-19b-3p-specific antagomir in JEV-infected mice reduces neuroinflammation and lethality. These findings suggest a new insight into the molecular mechanism of the JEV-induced inflammatory response and provide a possible therapeutic entry point for treating viral encephalitis.

## INTRODUCTION

Japanese encephalitis (JE) is endemic in most countries of South and East Asia and the Western Pacific, and infection with Japanese encephalitis virus (JEV) is the most common cause of viral encephalitis in children in the region (1, 2). JEV belongs to the Flaviviridae family of viruses, which also include, dengue, Murray Valley encephalitis, West Nile, and Zika viruses (2–4). A recent systemic review of data from 24 countries where JE is endemic reported a global estimate of JE incidence of roughly 68,000 cases per year, half of which occur in mainland China (5, 6). Young children aged <10 years have a greater risk of severe disease and associated residual neurologic deficit and a higher mortality rate (7).

JE is symbolized by profound neuronal cell damage along with substantial activation of glial cells, including microglia and astrocytes (8). During infection, JEV invades the central nervous system (CNS) and triggers a robust inflammatory response, resulting in increased levels of cytokines such as tumor necrosis factor alpha (TNF-α), interleukin-6 (IL-6), IL-1β, chemokine (C-C motif) ligand 5 (CCL5), and monocyte chemoattractant protein 1 (also called CCL2) in the cerebrospinal fluid (9). The increased levels of inflammatory mediators appear to play a defensive role or to commence an irreversible inflammatory response leading to neuronal cell death (9). Microglia and astrocytes are the major CNS-resident cells and represent critical effectors of CNS inflammation (10, 11). Microglia are considered the CNS professional macrophages due to their phenotypes and reactivity following injury and inflammation (12). For this reason, most of the studies on innate immune responses in the CNS have focused on microglia, which express a variety of pattern recognition receptors (11, 13, 14). The involvement of other cell types in these responses has often been ignored. However, recent evidence suggests that astrocytes also express a wide range of pattern recognition receptors and have a complex, dual role in the regulation of the cerebral immune response (15–20). Astrocyte-derived inflammatory responses play a critical role in neurological diseases (21–23). It has been reported that astrocytes can be directly infected by JEV and act as a reservoir of the virus (10, 24). The production of various proinflammatory mediators has been implicated in astrocytes activated following JEV infection (25, 26). Since astrocytes contribute the most abundant cell type in the brain, any disruption in the normal function of astrocytes can have drastic consequences on brain function (27).

MicroRNAs (miRNAs) are critical regulators of gene expression that utilize sequence complementarity to bind to and modulate the stability or translation efficiency of target mRNAs (28). Recent studies have revealed that miRNAs participate in various biological processes such as organogenesis, cellular proliferation and differentiation, apoptosis, innate and adaptive immunity, inflammation, and tumorigenesis (29–33). Accumulating evidence also suggests a decisive role for miRNAs in various neuroinflammatory diseases (34, 35), including viral encephalitis (36). For example, miR-155 regulates inflammatory cytokine production in human dendritic cells following lipopolysaccharide stimulation of these cells (37). miR-32 plays a pivotal role in human microglia activation following HIV infection (38). Other miRNAs, including miR-155 and miR-29b, are also reported to activate inflammatory cytokine production in microglia after JEV infection (39, 40). Critical roles for miR-181 and miR-146a in astrocyte-mediated inflammation have also been described (41, 42). We have also previously reported a role for miR-206 in lipopolysaccharide-mediated inflammatory cytokine production in human astrocytes (43).

Studies focusing on the role of miRNAs in JEV-mediated inflammation remain far behind other studies. Only three miRNAs, miR-155, miR-29b, and miR-146a, are reported to involve the production of inflammatory cytokines and suppression of the host innate immune response in microglia after JEV infection (39, 40, 44). However, it is relatively unknown how astrocytic miRNAs regulate JEV-induced inflammation. Recently, we reported the role of miR-15b in regulating JEV-induced expression of inflammatory cytokines in both astrocytes and microglia (36), suggesting that astrocytes are as important as microglia in regulating JEV-mediated inflammation.

In the present study, we found that miR-19b-3p is involved in regulating the JEV-induced inflammatory response *in vitro* and *in vivo* and that miR-19b-3p enhances the production of inflammatory cytokines in cultured cells and mouse brain tissues. Further investigations revealed that miR-19b-3p augments the inflammatory response via targeting ring finger protein 11 (RNF11), a negative regulator of nuclear factor kappa B (NF-κB) activity. *In vivo* treatment of JEV-infected mice with antagomir-19b-3p reduces overall neuroinflammation and improves survival rate.

## MATERIALS AND METHODS

### Virus isolation and titration.

Postnatal 3- to 4-day-old suckling BALB/c mice were infected with the P3 strain of JEV. Upon onset of clinical manifestations of JEV (poor brain response, limb paralysis, and whole-body tremor), animals were killed, and brains were excised. Brain homogenate prepared in Dulbecco's modified Eagle's medium (DMEM) was centrifuged at 10,000 × *g* to remove cellular debris. The resultant suspension was filtered through 0.22-μm-pore-size sterile filters to obtain a viral suspension. Immediately, aliquots of filtered virus suspension were stored at −80°C until further use. The virus titer was determined by plaque formation assay on baby hamster Syrian kidney (BHK-21) cells as described previously (45).

### Cell culture and treatment.

U251 cells (human astrocytoma cell line) and BV2 cells (mouse microglia cell line) were cultured and maintained in DMEM that was supplemented with 10% (vol/vol) heat-inactivated fetal bovine serum, 100 U/ml penicillin, and 100 mg/ml streptomycin sulfate at 37°C in a 5% CO_2_ atmosphere. U251 or BV2 cells were seeded in 6- or 12-well plates and grown to 80% confluence. Nonadherent cells were removed by washing with nonsupplemented DMEM prior to further treatment. Cells were subsequently transfected with RNAs and/or plasmids using Lipofectamine 2000 (Invitrogen). After 24 h, cell medium was removed, and cells were infected with JEV at a multiplicity of infection (MOI) of 5 for the times mentioned in the figure legends.

### Reagents.

Mouse monoclonal antibodies against JEV NS5 were generated in our laboratory. The following commercially obtained antibodies were used: rabbit monoclonal anti-RNF11 (for human RNF11; Abcam), rabbit polyclonal anti-RNF11 (for mouse RNF11; ABclonal Technology), rabbit polyclonal anti-NF-κB p65 (ABclonal Technology), rabbit polyclonal anti-lamin A (ABclonal Technology), mouse monoclonal anti-glyceraldehyde-3-phosphate dehydrogenase (GAPDH) (ABclonal Technology), and horseradish peroxidase-conjugated anti-mouse and anti-rabbit IgG secondary antibodies (Boster).

miR-19b-3p mimics (double-stranded RNA oligonucleotides), inhibitors (single-stranded chemically modified oligonucleotides), and control oligonucleotides were commercially purchased from GenePharma. Their sequences were as follows: miR-19b-3p mimics, 5′-UGUGCAAAUCCAUGCAAAACUGA-3′ (forward) and 5′-AGUUUUGCAUGGAUUUGCACAUU-3′ (reverse); mimic controls, 5′-UUCUCCGAACGUGUCACGUTT-3′ (forward) and 5′-ACGUGACACGUUCGGAGAATT-3′ (reverse); miR-19b-3p inhibitors, 5′-UCAGUUUUGCAUGGAUUUGCACA-3′; inhibitor controls, 5′-CAGUACUUUUGUGUAGUACAA-3′. Cholesterol-conjugated and chemically modified miR-19b-3p inhibitors (antagomir-19b-3p) were synthesized by GenePharma. The sequence of antagomir-19b-3p is 5′-U_s_G_s_UCAGUUUUGCAUGGAUUUGCACAG_s_C_s_U_s_A_s_-Chol-3′. In this sequence, the subscript “s” represents a phosphorothioate linkage, and “Chol” represents cholesterol linked through a hydroxyprolinol linkage. All nucleotides are 2′-*O*-methyl (2′-OMe) modified.

### Constructs and plasmids.

The psiCheck-2 dual-luciferase reporter vector (Promega) harboring the 3′ untranslated region (UTR) of RNF11 inserted into the XhoI and PmeI restriction sites at the 3′ end of Renilla luciferase was used to check the effect of miR-19b-3p on Renilla luciferase activity. The 3′ UTR of RNF11 was amplified from U251 cell genomic DNA with specific primers (forward, 5′-CCCTCGAGTGTGTAAAGTGTTAACAGCAGC-3′; reverse, 5′-GGGTTTAAACCTCAGTTTGCTTACAAGCTAGGA-3′). The psiCheck-2 mutant RNF11 3′ UTR construct was generated by inducing a point mutation using the overlap extension PCR method. Primers used for overlap extension PCR were as follows: forward, 5′-AAGCTTGTGCTAACAGTTCT-3′; reverse, 5′-AAAGAACTGTTAGCACAAGC-3′. All constructs were verified by sequencing.

### RNA interference.

A small interfering RNA (siRNA) corresponding to the RNF11 mRNA sequence (5′-GAGCCAGGGAUUAGGAAUUTT-3′; siRNF11), which was used to inhibit endogenous RNF11 expression, and a negative-control siRNA (5′-UUCUCCGAACGUGUCACGUTT-3′), which exhibited no downregulation of any human genes, were synthesized by GenePharma. Transfection was performed with Lipofectamine 2000 (Invitrogen). Cells were transfected with 50 nM each siRNA.

### Dual-luciferase reporter assays.

U251 cells were cotransfected with 100 ng of the constructed luciferase reporter plasmid along with miR-19b-3p mimics, inhibitors, or controls (final concentration, 50 nM). After 24 h of incubation, Renilla and firefly luciferase activities were measured using a dual-luciferase reporter assay system (Promega). The data are expressed as relative Renilla luciferase activity normalized to the value of firefly luciferase and are representative of three independent experiments.

### JEV infection and antagomir-19b-3p administration.

Adult C57BL/6 mice (8 weeks old) were obtained from the Hubei Provincial Center for Disease Control and Prevention, Wuhan, China. For the antagomir studies, mice were randomly assigned to three groups: group 1, control group (phosphate-buffered saline [PBS]); group 2, JEV-infected and antagomir negative-control-treated group (JEV-NC); and group 3, JEV-infected and antagomir-19b-3p-treated group (JEV-antagomir). Mice in groups 2 and 3 were inoculated intracranially with 100 PFU of the JEV P3 strain in 20 μl of PBS (36), whereas mice belonging to group 1 were injected intracranially with an equal volume of PBS. At 24 h postinfection, the antagomir negative control and antagomir-19b-3p (60 mg/kg body weight) were administered intravenously in mice of groups 2 and 3, respectively, for two consecutive days (36, 39). On day 6 postinfection, mice infected with JEV developed signs of acute encephalitis. The mice were euthanized, and brain samples were collected for further studies. The remaining mice were monitored daily to assess behavior and mortality. All animal experiments were performed according to the National Institutes of Health *Guide for the Care and Use of Laboratory Animals*(46), and the experimental protocols were approved by the Research Ethics Committee of the College of Veterinary Medicine, Huazhong Agricultural University, Hubei, Wuhan, China (number 42000600012034).

### RNA extraction and quantitative real-time PCR.

Total RNA was extracted using TRIzol reagent (Invitrogen), and 1 μg of RNA was used to synthesize cDNA using a first-strand cDNA synthesis kit (Toyobo). Quantitative real-time PCR was performed using a 7500 real-time PCR system (Applied Biosystems) and SYBR green PCR master mix (Toyobo). Data were normalized to the level of β-actin expression in each sample. Primers were as follows: RNF11, 5′-ACATCTCCCTGCTTCACGAG-3′ (forward) and 5′-GGGTGGTAGACTGGAACTGG-3′ (reverse); human β-actin, 5′-AGCGGGAAATCGTGCGTGAC-3′ (forward) and 5′-GGAAGGAAGGCTGGAAGAGTG-3′ (reverse); mouse β-actin, 5′-CACTGCCGCATCCTCTTCCTCCC-3′ (forward) and 5′-CAATAGTGATGACCTGGCCGT-3′ (reverse); human CCL5, 5′-CTGTCATCCTCATTGCTACTGC-3′ (forward) and 5′-ATGTACTCCCGAACCCATTTCT-3′ (reverse); mouse CCL5, 5′-TGCCCACGTCAAGGAGTATTTC-3′ (forward) and 5′-AACCCACTTCTTCTCTGGGTTG-3′ (reverse).

To quantify mature miRNA expression, a commercial bulge-loop miRNA reverse transcription-PCR (RT-PCR) detection method was used. Briefly, 1 μg of total RNA was used as the template and reverse transcribed using an miR-19b-3p-specific RT primer (5′-GTCGTATCCAGTGCAGGGTCCGAGGTATTCGCACTGGATACGACTCAGTT-3′). The resulting cDNA was used for quantitative real-time PCR with a universal reverse primer (5′-GTGCAGGGTCCGAGGT-3′) and an miR-19b-3p-specific forward primer (5′-TGTGCAAATCCATGCAAAACTGA-3′). Similarly, U6 small nuclear RNA was quantified using its reverse primer (5′-ATGGAACGCTTCACGAAT-3′) for the reverse transcription reaction and the forward primer (5′-TCGGCAGCACATATACTAA-3′) and reverse primer for quantitative real-time PCR. Amplification was performed for 2 min at 50°C and 10 min at 95°C, followed by 40 cycles of 95°C for 15 s, 60°C for 15 s, and 72°C for 30 s. The relative expression levels of miRNAs were normalized to the level of internal control U6 small nuclear RNA within each sample using the 2^−ΔΔ*CT*^ (where *C_T_* is threshold cycle) method. Expression was then standardized to the miRNA levels in mock-infected or control miRNA-treated cells.

### Immunoblotting.

Total cellular lysates or mouse brain tissue lysates were prepared using radioimmunoprecipitation assay buffer (Sigma) containing protease inhibitors (Roche). Cytosolic and nuclear extracts were prepared using NE-PEP Nuclear and Cytoplasmic Extraction Reagent (Thermo Scientific). Protein concentrations were determined using a bicinchoninic acid (BCA) protein assay kit (Thermo Scientific). Equal protein quantities were separated by SDS-PAGE and transferred to a polyvinylidene fluoride membrane (Millipore) using a Mini Trans-Blot Cell (Bio-Rad). Blots were probed with the relevant antibodies, and proteins were detected using enhanced chemiluminescent (ECL) reagent (Thermo Scientific).

### Plaque assay.

U251 cells were transfected with miR-19b-3p mimics, inhibitors, or their controls (final concentration, 50 nM) for 24 h and subsequently infected with JEV at an MOI of 5. At 12, 24, and 36 h postinfection, cell supernatants were harvested, serially diluted, and then used to inoculate monolayers of U251 cells. After removal of unbound JEV virus particles, U251 cells were further incubated for 3 to 5 days, and plaques were identified. The visible plaques were counted, and viral titers were calculated. All data are expressed as the means of triplicate samples.

### ELISA.

The culture supernatants were collected from the treated cells at the time points mentioned in the figure legends and stored at −80°C. The protein levels of TNF-α, IL-6, and IL-1β in cell cultures or mouse brain tissue lysates were determined by enzyme-linked immunosorbent assay (ELISA) kits (eBioscience) according to the manufacturer's instructions.

### H&E staining, IHC, and TUNEL assay.

Treated mice were anesthetized with ketamine-xylazine (0.1 ml/10 g body weight) and perfused with PBS followed by 4% paraformaldehyde. Brain tissues were removed and paraffin embedded for coronal sections. The sections were used for hematoxylin-eosin H&E staining, immunohistochemistry (IHC), and terminal deoxynucleotidyl transferase dUTP nick end labeling (TUNEL) assays as described previously (36). For IHC, sections were incubated overnight at 4°C with primary antibodies against glial fibrillary acidic protein (GFAP) (Dako) or ionized calcium binding adapter molecule 1 (IBA-1) (Wako). After being washed, slides were incubated with appropriate secondary antibodies and washed, and diaminobenzidine (DAB; Vector Laboratories) was utilized for color development. For the TUNEL assay, an *in situ* cell death detection kit (Roche) was used according to the manufacturer's instructions.

### Statistical analysis.

All experiments were repeated at least three times with similar results. Analyses were conducted using GraphPad Prism, version 5 (GraphPad Software, San Diego, CA). Results are expressed as means ± standard deviations (SD). Data were compared by two-way analysis of variance with subsequent *t* tests using a Bonferroni posttest for multiple comparisons or with the Student *t* test. For all tests, a *P* value of <0.05 was considered significant.

## RESULTS

### Upregulation of miR-19b-3p upon JEV infection.

**FIG 1:**
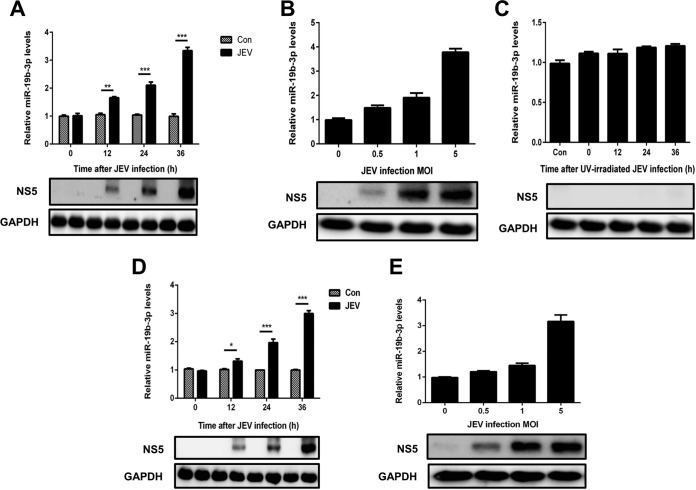
miR-19b-3p expression is upregulated after JEV infection. (A and B) U251 cells were infected with JEV at an MOI of 5 for the indicated times (A) or at the indicated MOIs for 36 h (B). (C) U251 cells were incubated with UV-inactivated JEV at an MOI of 5 for 36 h. (D and E) BV2 cells were infected with JEV at an MOI of 5 for the indicated times (D) or at the indicated MOIs for 36 h (E). The levels of miR-19b-3p were detected by quantitative real-time PCR (upper panels). Western blotting was performed to examine the expression of JEV NS5 protein (lower panels). GAPDH expression was verified as a loading control. All data are representative of at least three independent experiments. *, *P* < 0.05; **, *P* < 0.01; ***, *P* < 0.001. Con, control.

To interrogate the aftermath of JEV infection on the miRNA profile, miRNA deep-sequencing analysis of JEV-infected U251 cells was performed. Our sequencing data indicated that a group of miRNAs were differentially regulated upon JEV infection. Of these miRNAs, miR-19b-3p was found to be statistically well upregulated upon viral infection, and it was expressed at high levels in the cells (unpublished data). Because miR-19b-3p was found to be engaged in the cytokine- and chemokine-mediated inflammatory pathway (47), it was selected for further characterization during JEV infection. The corroboration of miR-19b-3p expression patterns in JEV-infected U251 cells was scrutinized by quantitative real-time PCR. The results revealed that miR-19b-3p was significantly upregulated in a time-dependent (Fig. 1A) and dose-dependent (Fig. 1B) manner. In contrast to these findings, UV-irradiated inactivated JEV infection failed to induce miR-19b-3p upregulation in U251 cells (Fig. 1C), suggesting possible involvement of miR-19b-3p in a JEV-induced inflammatory response in brain astrocytes. We also investigated the expression of miR-19b-3p in JEV-infected microglial BV2 cells (Fig. 1D and E). The results were similar to those observed in JEV-infected U251 cells. These results strongly demonstrate that miR-19b-3p expression is upregulated after JEV infection.

### Effects of miR-19b-3p on the JEV-triggered inflammatory cytokine production.

**FIG 2:**
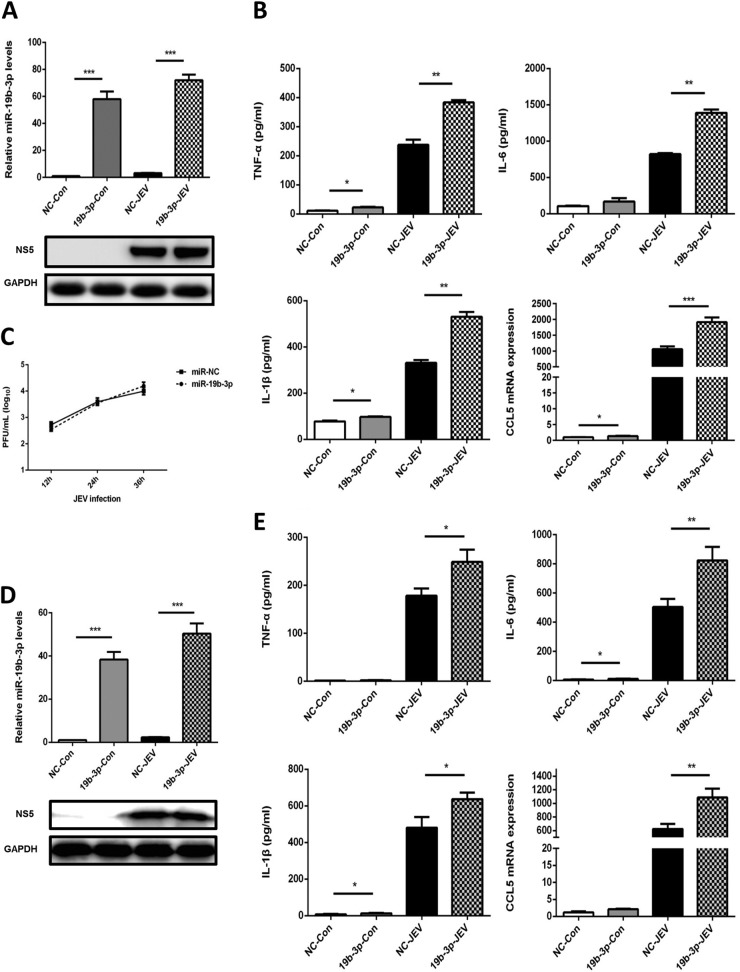
miR-19b-3p enhances JEV-mediated production of inflammatory cytokines. (A) U251 cells were transfected with miR-19b-3p mimics or control oligonucleotides (final concentration, 50 nM) for 24 h and then either left uninfected or infected with JEV at an MOI of 5 for 36 h. The level of miR-19b-3p was analyzed by quantitative real-time PCR and normalized to the U6 level. ***, *P* < 0.001. (B) The protein levels of TNF-α, IL-6, and IL-1β were analyzed by ELISA. Data represent means ± SD from three independent experiments performed in duplicate. *, *P* < 0.05; **, *P* < 0.01. CCL5 mRNA levels were determined by quantitative real-time PCR and normalized to the expression of β-actin in each sample. Data represent means ± SD from three independent experiments. *, *P* < 0.05; ***, *P* < 0.001. (C) The transfected U251 cells were infected with JEV at an MOI of 5. Cells were collected at the indicated time points, and titers of infectious virus in the culture supernatants were determined by plaque assay. The data represent three independent experiments with identical results. (D) BV2 cells were transfected with miR-19b-3p mimics or control oligonucleotides (final concentration, 50 nM) for 24 h and then either left uninfected or infected with JEV at an MOI of 5 for 36 h. The level of miR-19b-3p was analyzed by quantitative real-time PCR and normalized to the U6 level. ***, *P* < 0.001. (E) The protein levels of TNF-α, IL-6, and IL-1β were analyzed by ELISA. Data represent means ± SD from three independent experiments performed in duplicate. *, *P* < 0.05; **, *P* < 0.01. CCL5 mRNA levels were determined by quantitative real-time PCR and normalized to the expression of β-actin in each sample. Data represent means ± SD from three independent experiments. **, *P* < 0.01.

**FIG 3:**
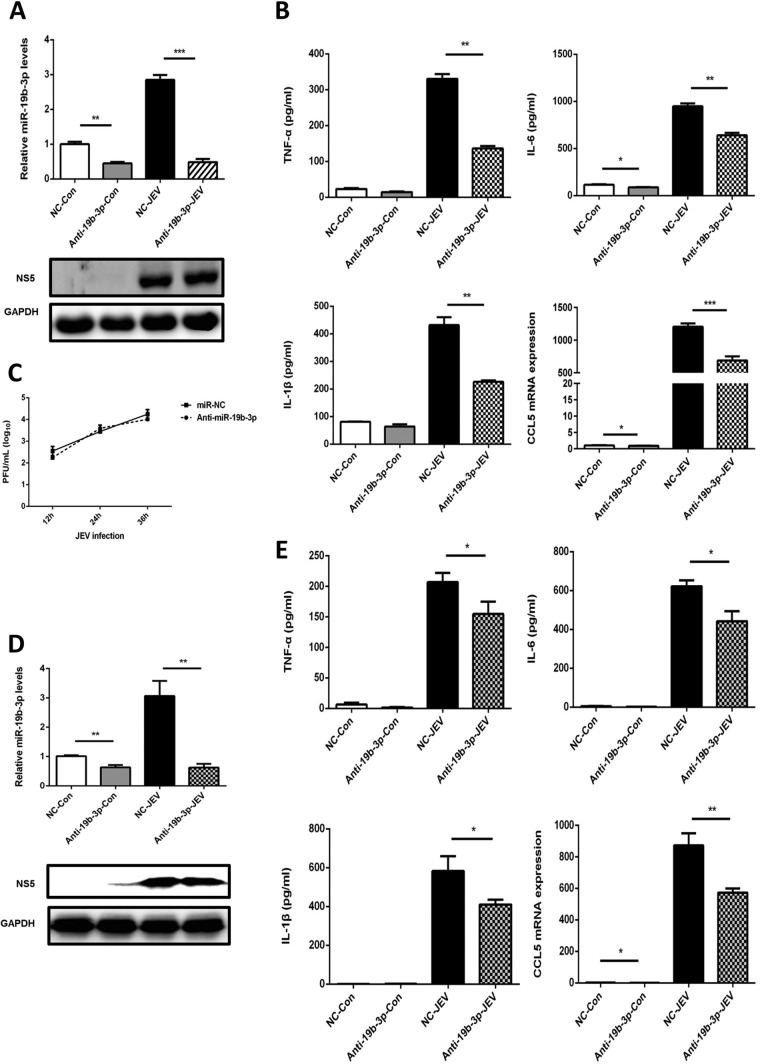
Inhibition of miR-19b-3p suppresses JEV-mediated production of inflammatory cytokines. (A) U251 cells were transfected with miR-19b-3p inhibitors or control oligonucleotides (final concentration, 50 nM) for 24 h and then either left uninfected or infected with JEV at an MOI of 5 for 36 h. The level of miR-19b-3p was analyzed by quantitative real-time PCR and normalized to the U6 level. **, *P* < 0.01; ***, *P* < 0.001. (B) The protein levels of TNF-α, IL-6, and IL-1β were analyzed by ELISA. Data represent means ± SD from three independent experiments performed in duplicate. *, *P* < 0.05; **, *P* < 0.01. CCL5 mRNA levels were determined by quantitative real-time PCR and normalized to the expression of β-actin in each sample. Data represent means ± SD from three independent experiments. *, *P* < 0.05; ***, *P* < 0.001. (C) The transfected U251 cells were infected with JEV at an MOI of 5. Cells were collected at the indicated time points, and titers of infectious virus in the culture supernatants were determined by plaque assay. The data represent three independent experiments with identical results. (D) BV2 cells were transfected with miR-19b-3p inhibitors or control oligonucleotides (final concentration, 50 nM) for 24 h and then either left uninfected or infected with JEV at an MOI of 5 for 36 h. The level of miR-19b-3p was analyzed by quantitative real-time PCR and normalized to the U6 level. **, *P* < 0.01. (E) The protein levels of TNF-α, IL-6, and IL-1β were analyzed by ELISA. Data represent means ± SD from three independent experiments performed in duplicate. *, *P* < 0.05. CCL5 mRNA levels were determined by quantitative real-time PCR and normalized to the expression of β-actin in each sample. Data represent means ± SD from three independent experiments. *, *P* < 0.05; **, *P* < 0.01.

JE is characterized by stimulation of resident glial cells and the excessive release of inflammatory cytokines, which leads to neuronal cell death (8, 9). To examine whether miR-19b-3p is involved in the JEV-mediated inflammatory process, the effect of miR-19b-3p on the regulation of inflammatory cytokine production after JEV infection was determined. First, we evaluated the consequences of synthetic miR-19b-3p mimics and inhibitors on the expression pattern of miR-19b-3p. miRNA mimics are double-stranded RNAs synthesized to simulate naturally occurring mature miRNAs, whereas inhibitors are chemically modified antisense single-stranded RNAs that curb the yielding of endogenous miRNAs by sequence complementarity. miRNA mimics and inhibitors can cause fluctuations in the levels of the physiological miRNAs. As expected, transfection of miR-19b-3p mimics increased miR-19b-3p levels significantly in mock- or JEV-infected glial cells at 36 h postinfection (Fig. 2A and D), whereas miR-19b-3p inhibitors diminished its levels (Fig. 3A and D). Interestingly, certain cytokines of JEV-induced neuroinflammation, such as TNF-α, IL-6, IL-1β, and CCL5 (9, 36), were found to be significantly upregulated upon transfection of miR-19b-3p mimics in both infected and uninfected U251 cells (Fig. 2B). In contrast, the inhibition of endogenous miR-19b-3p significantly repressed JEV-triggered cytokine production (Fig. 3B). Furthermore, we found that treatment of miR-19b-3p mimics or inhibitors did not produce any antiviral activity in JEV-infected U251 cells as viral titers were similar to those in control cells (Fig. 2C and 3C). The effects of miR-19b-3p mimics and inhibitors on inflammatory cytokine production were also examined in BV2 cells, and the results were analogous to those observed in U251 cells (Fig. 2E and 3E). Thus, these data indicate that miR-19b-3p participates in regulating JEV-mediated inflammation.

### RNF11 is a potential target of miR-19b-3p.

**FIG 4:**
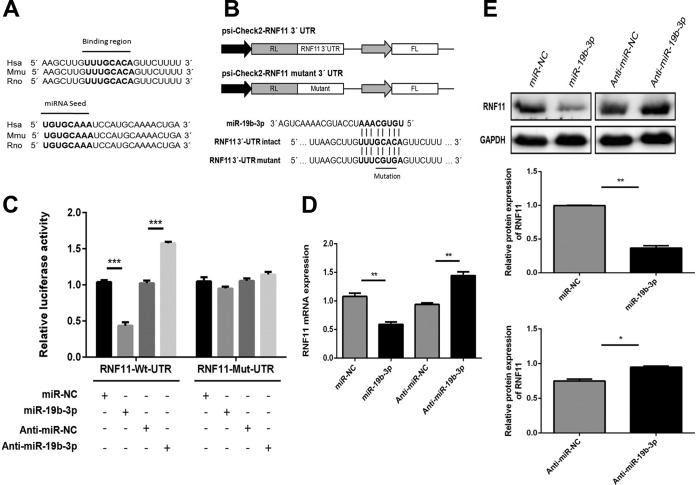
miR-19b-3p directly targets RNF11. (A) Conservation of the miR-19b-3p target sequence in RNF11 among different species (upper panel), and conservation of the sequence of miR-19b-3p among different species (lower panel). Hsa, Homo sapiens (human); Mmu, Mus musculus (mouse); Rno; Rattus norvegicus (rat). (B) Schematic diagram showing dual-luciferase reporter constructs harboring the 3′ UTR of RNF11 with putative miR-19b-3p binding site. The lower panel shows the alignment of miR-19b-3p and its target site in the 3′ UTR of RNF11. Four mutated nucleotides of the target site are underlined. RL, Renilla luciferase; FL, firefly luciferase. (C) U251 cells were cotransfected with miR-19b-3p mimics, miR-19b-3p inhibitors, or the corresponding control oligonucleotide (final concentration, 50 nM) together with a wild-type or mutated RNF11 3′ UTR dual-luciferase reporter plasmid, and Renilla luciferase activity was measured and normalized to firefly luciferase activity after 24 h. (D and E) U251 cells were transfected with miR-19b-3p mimics, miR-19b-3p inhibitors, or the corresponding control oligonucleotide (final concentration, 50 nM), and then RNF11 mRNA (C) and protein levels (D) were determined after 24 h by quantitative real-time PCR and immunoblotting, respectively. Data represent means ± SD from three independent experiments. *, *P* < 0.05; **, *P* < 0.01; ***, *P* < 0.001. Protein levels were quantified with immunoblot scanning and normalized to the amount of GAPDH expression.

A previous study has reported RNF11 as a potential target for miR-19b-3p (47). The sequences of miR-19b-3p and its target site in the 3′ UTR of RNF11 were aligned with those from different species, and these sequences are shown to be highly conserved among species (Fig. 4A). These findings were determined using publicly available miRNA target prediction algorithms that include TargetScan, Pictar, and miRanda. To determine if RNF11 mRNA is indeed repressed by miR-19b-3p in the context of virus infection, a dual-luciferase reporter plasmid containing a putative binding site for miR-19b-3p and a mutant construct harboring the miR-19b-3p seed region with a 4-bp mutation were generated (Fig. 4B). A marked reduction in luciferase activity was observed in U251 cells cotransfected with miR-19b-3p mimics and the RNF11 wild-type 3′ UTR carrying a binding site, whereas significantly increased luciferase activity was detected following application of miR-19b-3p inhibitors (Fig. 4C). Moreover, the 4-bp mutation of in the miR-19b-3p seed region led to a complete abrogation of the negative effect of miR-19b-3p on expression of RNF11 3′ UTR reporter constructs (Fig. 4C). These results demonstrate that the nucleotide sequence in the 3′ UTR of RNF11 is a potential miR-19b-3p targeting site. To further substantiate that RNF11 is indeed a target of miR-19b-3p, endogenous RNF11 expression was determined in U251 cells treated with miR-19b-3p mimics or inhibitors. As shown in Fig. 4D and E, the ectopic expression of miR-19b-3p significantly repressed RNF11 mRNA and protein levels, whereas miR-19b-3p inhibitors restored RNF11 expression, indicating that RNF11 expression could be squelched by miR-19b-3p via mRNA decay and translational suppression. Thus, these data suggest that RNF11 is a direct target of miR-19b-3p and that its expression is modulated by miR-19b-3p.

### JEV infection downregulates RNF11 expression.

**FIG 5:**
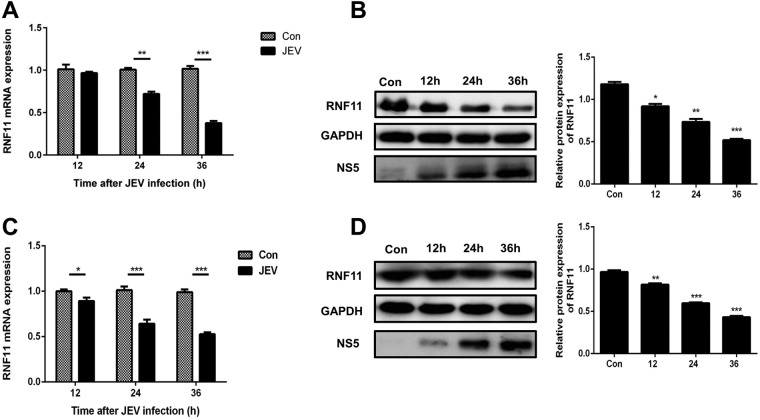
RNF11 expression is downregulated after JEV infection. (A) U251 cells were infected with JEV at an MOI of 5 for the indicated times, and then RNF11 mRNA expression levels were determined by quantitative real-time PCR. (B) U251 cells were infected with JEV at an MOI of 5 for the indicated times, and then RNF11 protein levels were determined with immunoblotting. (C and D) BV2 cells were infected with JEV at an MOI of 5 for the indicated times, and then RNF11 mRNA (C) and protein (D) expression levels were determined by quantitative real-time PCR and immunoblotting, respectively. Data represent means ± SD from three independent experiments. *, *P* < 0.05; **, *P* < 0.01; ***, *P* < 0.001. Protein levels were quantified with immunoblot scanning and normalized to the amount of GAPDH expression.

To study the effect of JEV infection on RNF11, the time-dependent expression patterns of RNF11 mRNA (Fig. 5A) and protein (Fig. 5B) in U251 cells following JEV infection were studied. Significant downregulation of RNF11 mRNA and protein levels at 12, 24, and 36 h postinfection was observed. Furthermore, RNF11 mRNA and protein expression levels were also determined in JEV-infected BV2 cells (Fig. 5C and D). The results were concordant with those in JEV-infected U251 cells. Thus, these data demonstrate that RNF11 expression is downregulated upon JEV infection.

### miR-19b-3p regulates JEV-induced inflammatory cytokine expression by targeting RNF11.

**FIG 6:**
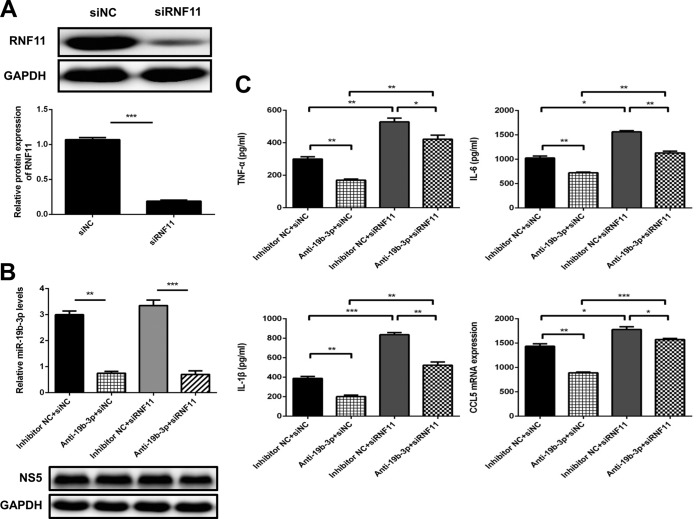
Regulation of JEV-induced production of inflammatory cytokines by miR-19b-3p is achieved through RNF11. (A) U251 cells were transfected with siRNF11 or nonspecific control siRNA (siNC) (final concentration, 50 nM) for 24 h, and then RNF11 protein levels were measured by immunoblotting. Protein levels were quantified by immunoblot scanning and normalized to the amount of GAPDH expression. (B) U251 cells were cotransfected with miR-19b-3p inhibitors or control oligonucleotides and siRNF11 or a nonspecific control siRNA (final concentration, 50 nM) for 24 h and then infected with JEV at an MOI of 5 for 36 h. The levels of miR-19b-3p were analyzed by quantitative real-time PCR and normalized to the U6 level. **, *P* < 0.01; ***, *P* < 0.001. (C) The protein levels of TNF-α, IL-6, and IL-1β were analyzed by ELISA. Data represent means ± SD from three independent experiments performed in duplicate. *, *P* < 0.05; **, *P* < 0.01; ***, *P* < 0.001. CCL5 mRNA levels were determined with quantitative real-time PCR and normalized to the expression of β-actin in each sample. Data represent means ± SD from three independent experiments. *, *P* < 0.05; **, *P* < 0.01; ***, *P* < 0.001.

To determine whether the observed effects of miR-19b-3p on inflammatory cytokine production in response to JEV infection were, at least partially, mediated through RNF11, we analyzed the effects of silencing RNF11 expression by siRNA in U251 cells. We confirmed that the siRNA significantly inhibited RNF11 protein expression in U251 cells (Fig. 6A).

The cells were cotransfected with miR-19b-3p inhibitors or control oligonucleotides and siRNF11 or a nonspecific control siRNA and then infected with JEV. First, we confirmed the expression of miR-19b-3p in the transfected cells (Fig. 6B). Knockdown of RNF11 significantly increased the production of inflammatory cytokines, which means that RNF11 silencing produces effects similar to those of miR-19b-3p overexpression (Fig. 6C). In line with previous data, JEV-induced expression of TNF-α, IL-6, IL-1β, and CCL5 was decreased by miR-19b-3p inhibitors. Importantly, silencing of RNF11 rescued the suppressive effect of miR-19b-3p inhibitors on these cytokines (Fig. 6C). These results suggest that silencing of RNF11 phenocopied the proinflammatory effect of miR-19b-3p and counteracted the effect of anti-miR-19b-3p.

### miR-19b-3p expression activates the NF-κB pathway in JEV-infected astrocytes.

**FIG 7:**
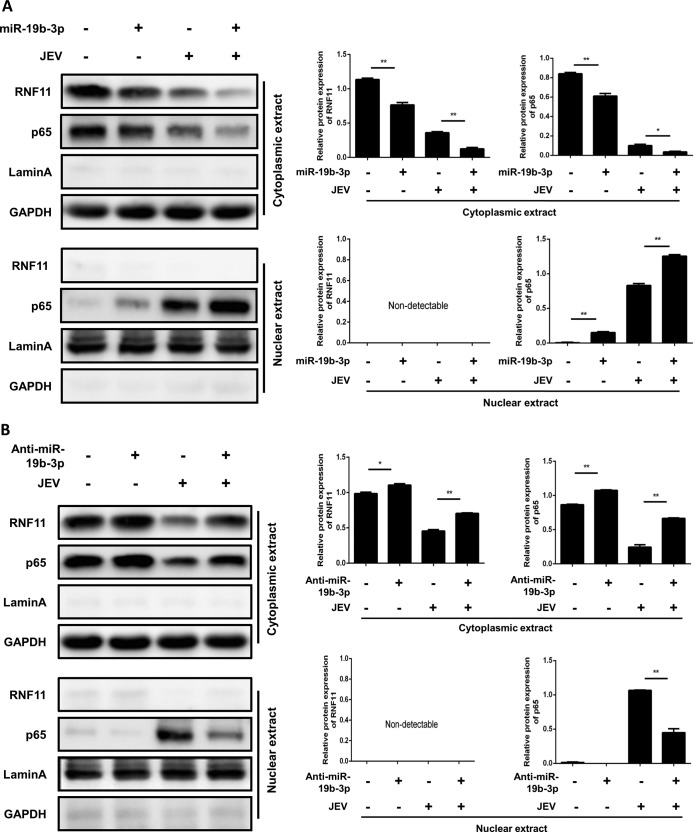
miR-19b-3p activates the NF-κB pathway in JEV-infected astrocytes. (A and B) U251 cells were transfected with miR-19b-3p mimics, inhibitors, or their control oligonucleotides (final concentration, 50 nM) for 24 h and then either left uninfected or infected with JEV at an MOI of 5 for 36 h. The cytosolic extracts (upper panel) and nuclear extracts (lower panel) were isolated and subjected to immunoblotting with antibodies against RNF11, NF-κB p65, lamin A, and GAPDH. Lamin A was used as a marker for nuclei. GAPDH and lamin A were used as the loading controls. Protein levels were quantified by immunoblot scanning and normalized to the amount of GAPDH or lamin A expression. *, *P* < 0.05; **, *P* < 0.01.

It has been well established that translocation of NF-κB from the cytoplasm to the nucleus is a key determinant of NF-κB activation (36, 48). Therefore, it was of interest to evaluate the effect of miR-19b-3p on NF-κB activation in JEV-infected astrocytes. Nuclear translocation of NF-κB (p65) was detected with immunoblotting. Transfection of miR-19b-3p mimics increased the translocation of NF-κB from the cytoplasm to the nucleus (Fig. 7A). In contrast, treatment of cells with miR-19b-3p inhibitors significantly inhibited the nuclear translocation of NF-κB in JEV-infected U251 cells (Fig. 7B). Thus, these findings demonstrated that miR-19b-3p appears to regulate inflammatory cytokine production by enhancing the activation of NF-κB signaling in JEV-infected astrocytes.

### miR-19b-3p activates NF-κB signaling via targeting RNF11 in JEV-infected astrocytes.

**FIG 8:**
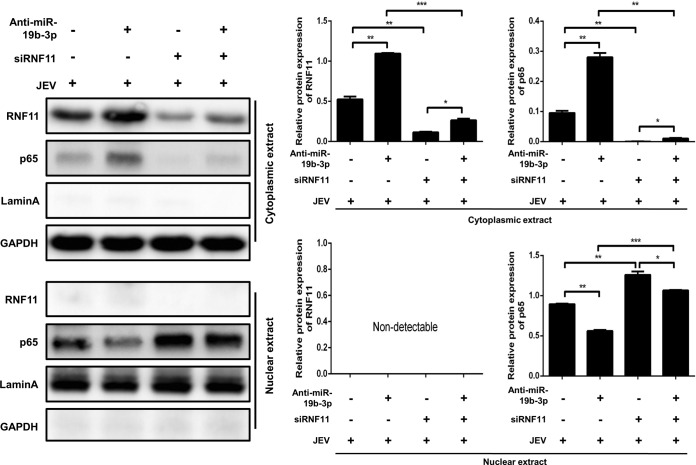
miR-19b-3p activates NF-κB signaling via targeting RNF11 in JEV-infected astrocytes. U251 cells were cotransfected with miR-19b-3p inhibitors or control oligonucleotides and siRNF11 or a nonspecific control siRNA (final concentration, 50 nM) for 24 h and then infected with JEV at an MOI of 5 for 36 h. The cytosolic extracts (upper panel) and nuclear extracts (lower panel) were isolated and subjected to immunoblotting with antibodies against RNF11, NF-κB p65, lamin A, and GAPDH. Lamin A was used as a marker for nuclei. GAPDH and lamin A were used as the loading controls. Protein levels were quantified by immunoblot scanning and normalized to the amount of GAPDH or lamin A expression. *, *P* < 0.05; **, *P* < 0.01; ***, *P* < 0.001.

To substantiate that miR-19b-3p is indeed involved in the regulation of NF-κB signaling through RNF11, U251 cells were cotransfected with miR-19b-3p inhibitors or control oligonucleotides and siRNF11 or a nonspecific control siRNA and subsequently infected with JEV. As expected, silencing of RNF11 significantly enhanced the accumulation of NF-κB in the nucleus, and these effects were concordant with miR-19b-3p overexpression (Fig. 8). Similar to our previous data, JEV-induced nuclear translocation of NF-κB was decreased by miR-19b-3p inhibitors. However, knockdown of RNF11 rescued the inhibitory effects of miR-19b-3p inhibitors on NF-κB activity (Fig. 8), suggesting that miR-19b-3p activates NF-κB activity via targeting RNF11.

### Antagomir-19b-3p treatment reduces neuroinflammation and lethality in JEV-infected mice.

To delineate the significance of miR-19b-3p in JEV-caused encephalitis *in vivo*, a mouse model for JEV infection was established. The antagomir-19b-3p, a chemically modified antisense oligonucleotide, was administered intravenously into mice at 24 and 36 h postinfection to knock down the expression of endogenous miR-19b-3p. This chemically modified antagomir can cross the blood-brain barrier via intravenous routes, as described previously (36, 39). Mice tolerated antagomir-19b-3p well, without showing any sign of discomfort or sickness. On day 6 postinfection, JEV-infected mice exhibited typical symptoms of JE that included limb paralysis, restriction of body movement, and ataxia. Mice were sacrificed, and brain samples were collected and processed for subsequent experiments.

**FIG 9:**
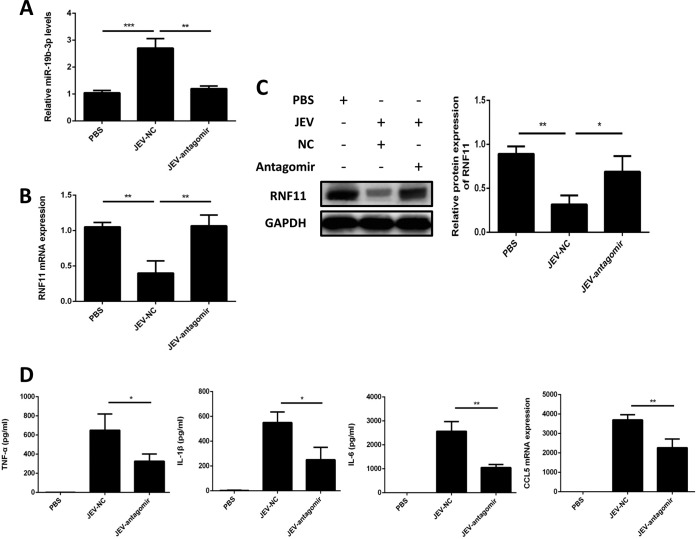
Antagomir-19b-3p treatment stimulates RNF11 expression and reduces inflammatory cytokine production in a mouse model of JEV infection. Mice were treated with antagomir negative control or antagomir-19b-3p (60 mg/kg body weight) after JEV infection, and brain samples were collected at day 6 postinfection. PBS, samples collected from PBS-challenged mice; JEV-NC, samples collected from JEV-infected mice treated with antagomir negative control; JEV-antagomir, samples collected from JEV-infected mice treated with antagomir-19b-3p. (A) The increase of miR-19b-3p expression was abrogated in JEV-infected mice by administration of antagomir-19b-3p. (B and C) Suppression of RNF11 was reversed by antagomir-19b-3p treatment in JEV-infected mice. RNF11 mRNA (B) and protein (C) levels were determined by quantitative real-time PCR and immunoblotting, respectively. Protein levels were quantified by immunoblot scanning and normalized to the amount of GAPDH expression. (D) Antagomir-19b-3p treatment decreased the production of inflammatory cytokines. The protein levels of TNF-α, IL-6, and IL-1β were analyzed by ELISA. CCL5 mRNA levels were determined by quantitative real-time PCR and normalized to the expression of β-actin. The effects of antagomir-19b-3p and the antagomir negative control were compared using two-tailed Student's *t* tests. Similar results were obtained in three mice. *, *P* < 0.05; **, *P* < 0.01; ***, *P* < 0.001.

Similar to our *in vitro* findings, brain tissues from JEV-infected mice demonstrated an inverse relationship between the expression patterns of miR-19b-3p and its target, RNF11; i.e., higher miR-19b-3p expression was correlated with a reduced level of RNF11 (Fig. 9A to C). In JEV-infected mice, treatment of antagomir-19b-3p caused a specific reduction in miR-19b-3p expression and rescued the alterations in RNF11 levels (Fig. 9A to C). Moreover, we observed that inflammatory cytokines, including TNF-α, IL-6, IL-1β, and CCL5, were significantly augmented in JEV-infected mice treated with the antagomir negative control, whereas these cytokine levels were attenuated upon antagomir-19b-3p treatment (Fig. 9D). Because inflammatory cytokines are responsible for neuropathological and cytotoxic conditions during JEV infection (8, 26), a diminished level of cytokines upon antagomir-19b-3p treatment could be favorable in reducing neuronal cell damage.

**FIG 10:**
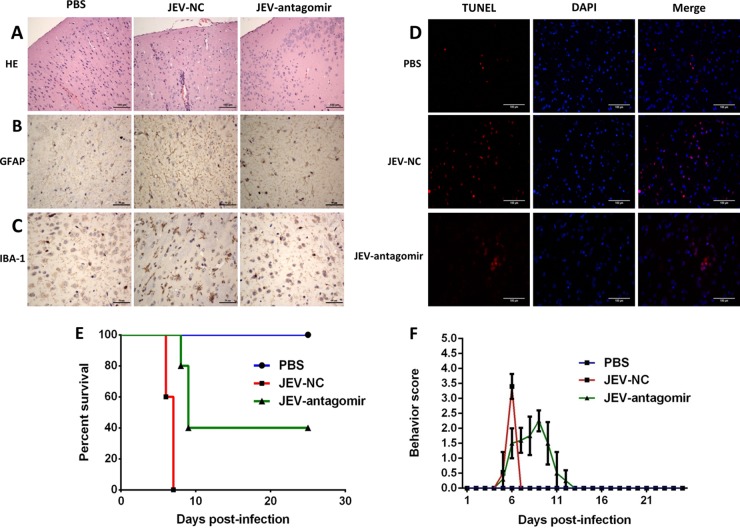
Antagomir-19b-3p treatment reduces neuroinflammation and improves the survival rate in JEV-infected mice. (A) Antagomir-19b-3p treatment ameliorates histopathological changes in JEV-infected mice. H&E staining of brain sections was performed to examine the pathological changes. Scale bar, 100 μm. (B and C) Antagomir-19b-3p treatment reduces astrocytosis and microgliosis. Sections of brain were analyzed by IHC staining. (B) Activation of astrocytes was detected by anti-GFAP antibody (B). Activation of microglia was detected by anti-IBA-1 antibody (C). Scale bar, 50 μm. (D) Antagomir-19b-3p treatment decreases neuronal cell damage. The apoptotic cells in the brain sections were stained using a TUNEL assay kit. Scale bar, 100 μm. The figures are a representative of three mice with similar results. (E) Antagomir-19b-3p treatment improves the survival rate. Survival of mice in each group was recorded for 25 days after JEV infection (100 PFU/mice) intracranially. Data were collected and are shown as Kaplan-Meier survival curves (*n* = 5 for each group). (F) Behavior score chart showing the gradual alleviation of suffering following JEV infection. Behavior scoring is as follows: 0, no restriction of movement, no frequent blinking, no body stiffening, no hind limb paralysis; 1, no restriction of movement, frequent blinking, no body stiffening, no hind limb paralysis; 2, restriction of movement, frequent blinking, no body stiffening, no hind limb paralysis; 3, restriction of movement, body stiffening, no hind limb paralysis; 4, restriction of movement, closed eyes, body stiffening, hind limb paralysis, body tremors.

On day 6 postinfection, brain sections from PBS- or JEV-challenged mice were examined for pathological variations. Brains of antagomir negative-control-treated mice presented the emblematic histopathological features of encephalitis, whereas pathological changes were ameliorated upon antagomir-19b-3p treatment (Fig. 10A). JEV infection is also associated with marked activation of astrocytes and microglia (26, 36). An aberrant increase in the number of astrocytes and microglia was detected by IHC using anti-GFAP and -IBA-1 antibody, respectively. Reactive astrocytosis and microgliosis were noticed in the antagomir negative-control-treated group, while antagomir-19b-3p treatment reduced the proliferation of these glial cells markedly (Fig. 10B and C). To examine whether antagomir-19b-3p can reduce neuronal cell damage, brain sections were subjected to a TUNEL assay. The number of dead cells was significantly lower with antagomir-19b-3p treatment in JEV-infected mice than in antagomir negative-control-treated mice (Fig. 10D). These findings indicate that antagomir-19b-3p treatment has the potential to abrogate the activation of astrocytes and microglia and to subsequently reduce neuronal cell death.

Given the role of antagomir-19b-3p in reducing neuroinflammation in JEV-infected mice, we assessed the potential of antagomir-19b-3p in protecting mice against JEV-associated lethality. All mice in the PBS groups survived during observation. A high mortality rate (100%) was observed in the antagomir negative-control-treated group on day 7 postinfection with JEV (Fig. 10E). In contrast, administration of antagomir-19b-3p protected mice from mortality within 7 days postinfection, and a 40% survival rate was recorded during the entire 25-day period of observation (Fig. 10E). Treatment with antagomir-19b-3p also caused improved behavioral signs in JEV-infected mice (Fig. 10F). Thus, our study demonstrates the therapeutic potential of antagomir-19b-3p in reducing JEV-induced neuroinflammation and lethality.

## DISCUSSION

Innate immunity is an imperative component of the CNS and depends mainly on resident microglia (49). However, evidence is emerging that the most populous type of glial cells of the CNS, the astrocytes, also engage in the local innate immune response stimulated by a variety of pathogen-derived molecular motifs (49). Astrocytes present an array of receptors associated with innate immunity, such as Toll-like receptors, double-stranded RNA-dependent protein kinase, nucleotide-binding oligomerization domains, mannose receptors, scavenger receptors, and components of the complement system (14, 16, 18, 19, 50). Upon activation, astrocytes are endowed with the capability to release inflammatory cytokines and enhance localized inflammation (51). The decisive role of astrocytes in regulating neuroinflammation has also been described by various *in vivo* studies (52, 53). The neurotropic viruses such as JEV, enterovirus 71, human immunodeficiency virus 1, herpes simplex virus 1, and vesicular stomatitis virus can replicate in astrocytes and, in turn, contribute to neuropathogenesis (54–58). Multiple miRNAs modulate host innate immune signaling pathways, which results in inflammatory responses (43, 59), but the roles of miRNAs in astrocyte-derived inflammatory responses are not fully understood. In this study, we showed that astrocytes expressed high levels of miR-19b-3p upon JEV infection. We also demonstrated that miR-19b-3p positively regulates the JEV-induced inflammatory response *in vitro* and *in vivo* via targeting RNF11, a negative regulator of NF-κB signaling (60).

miR-19b-3p belongs to the miR-17/92 cluster of miRNAs, and this miRNA cluster has been found to have divergent roles in the development of tumors and other diseases (61, 62). Genetic anatomization of the relative contribution of the individual miRNAs of this cluster has demonstrated that miR-19 recapitulated on its own the oncogenic effects of the full cluster (63). In addition to its oncogenic effects, the miR-19 regulon is reported to control NF-κB signaling by targeting members of the ubiquitin-editing protein complex in the cells stimulated with purified bacterial product (47), suggesting that targeting this miRNA regulon could regulate the activity of NF-κB signaling in inflammation. To date, no study has reported the role of the miR-19 regulon in the context of any virus-mediated inflammatory response. Since our deep-sequencing data revealed that miR-19b-3p is upregulated after JEV infection, we hypothesized that miR-19b-3p may have a crucial role in regulating the JEV-induced inflammatory response. In the present study, we found that miR-19b-3p is upregulated in JEV-infected U251 and BV2 cells and that it reinforces the production of inflammatory cytokines such as TNF-α, IL-6, IL-1β, and CCL5. This upsurge of inflammatory cytokines was achieved through suppression of RNF11, a direct target of miR-19b-3p. Interestingly, we also observed that overall expression levels of inflammatory cytokines in BV2 cells were lower than those in U251 cells, suggesting that astrocytes may react more effectively than microglia in regulating the miR-19b-3p-mediated inflammatory response upon JEV infection.

RNF11 is a 154-amino-acid protein which exhibits differential expression in cancer as well as in Parkinson's disease (64, 65). RNF11 contains a RING H2 finger domain in its C terminus, which is a characteristic feature of an E3 ubiquitin ligase (66). RNF11 enhances transforming growth factor β (TGF-β) signaling via interacting with Smurf2 and Smad4 (67, 68). It is also an essential component of the A20 ubiquitin-editing complex and can negatively regulate NF-κB signaling in human monocytic cell lines (69). RNF11 is also differentially expressed in the neurons and glial cells (70); however, the exact functions of RNF11 in the nervous system are poorly understood. On the basis of the characterization of RNF11 in monocytic cell lines (69), its role in Parkinson's disease (65), its differential expression in neurons and glial cells (70), and our confirmed analysis of the upregulation of miR-19b-3p after JEV infection, we hypothesized that RNF11 may also have a role in inducing the JEV-mediated inflammatory response. Here, we found that RNF11 expression is significantly downregulated in JEV-infected glial cells. Overexpression of miR-19b-3p markedly diminished RNF11 mRNA and protein levels, whereas treatment with miR-19b-3p inhibitors restored RNF11 expression. We also determined that silencing of RNF11 by siRNAs significantly enhanced inflammatory cytokine production. Moreover, silencing of RNF11 rescued the inhibitory effect of miR-19b-3p inhibitors on JEV-induced expression of inflammatory cytokines. Taken together, our findings demonstrate that miR-19b-3p positively regulates JEV-induced inflammatory cytokine production by targeting RNF11.

NF-κB activity is indispensable for peripheral immune cell survival, and appropriate regulation of NF-κB signaling is critical for coordinating a normal immune process (71). Persistent activation of NF-κB signaling is known to promote inflammation and inflammatory diseases (71, 72). Notably, suppression of NF-κB activity has been reported to reduce neuroinflammation and cell death in animal models of Alzheimer's disease, Parkinson's disease, and ischemia (73–75). A number of studies have also demonstrated miRNA-mediated regulation of NF-κB signaling (43, 76); however, its regulation through an interplay between miRNAs and proteins belonging to the ubiquitin-editing complex, including RNF11, is poorly understood. miR-29b has been documented to intensify NF-κB signaling by targeting tumor necrosis factor alpha-induced protein 3, a member of the ubiquitin-editing complex (40), suggesting that the regulation of the ubiquitin-editing complex is under the control of miRNAs. Therefore, it was of interest to evaluate the effect of miR-19b-3p in regulating JEV-induced NF-κB activity. We found that treatment of cells with miR-19b-3p mimics enhances the translocation of NF-κB from the cytoplasm to the nucleus, whereas miR-19b-3p inhibitors hinder the nuclear translocation of NF-κB. In addition, siRNA-induced knockdown of RNF11 escalates the accumulation of NF-κB in the nucleus, where it can stimulate the transcription of inflammatory cytokine genes. Thus, these findings illustrate that JEV-induced miR-19b-3p potentiates NF-κB signaling through RNF11.

Extensive gliosis is a characteristic feature of JEV infection, which results in the inordinate production of inflammatory cytokines and subsequently leads to neuronal cell damage (8, 26). Therefore, suppression of an undue inflammatory response can potentially terminate the progression of events leading to morbidity and mortality caused by JEV infection and seems to be a remedy against JEV infection. In this regard, a JEV-infected mouse model was established to substantiate the effects of miR-19b-3p *in vivo*. In JEV-infected mouse brain tissues, miR-19b-3p showed a reciprocal expression pattern with RNF11, which further supports a functional interplay between miRNA and mRNA *in vivo*. Antagomir-19b-3p treatment in an initial phase of virus infection has inhibitory effects on cytokine production in JEV-infected mice; it can inhibit the activation of astrocytes and microglia and reduce neuronal cell damage. After antagomir-19b-3p treatment, 40% of JEV-infected mice became asymptomatic and survived for at least 25 days postinfection, whereas the antagomir negative-control-treated mice remained weak and died within 7 days. Our results indicate that JEV-induced neuroinflammation is the key factor in predicting the progression of JEV infection and that suppression of inflammatory cytokines in the acute phase is imperative to improve survival.

In summary, this study provides evidence that miR-19b-3p acts as a positive regulator of JEV-induced neuroinflammation by enhancing NF-κB signaling, resulting in increased production of TNF-α, IL-6, IL-1β, and CCL5. To the best of our knowledge, our data suggest for the first time that miR-19b-3p-mediated regulation of RNF11 participates in the induction of an inflammatory response in the context of viral infections. Therefore, inhibition of miR-19b-3p may be an intriguing approach for the advancement of curative interventions. Moreover, this study may also provide insight into the use of miRNA-based therapeutics against other neuroinflammatory diseases.
